# Safety and efficacy of Modified Delorme's procedure for rectal prolapse

**DOI:** 10.6026/973206300211585

**Published:** 2025-06-30

**Authors:** Nandram Dhakariya, Bhupesh Kushram, Bharti Badlani, Anjulika Sachan, Abhay Kumar

**Affiliations:** 1 Department of General Surgery, Chhindwara Institute of Medical Sciences, Chhindwara, Madhya Pradesh, India; 2 Department of Ophthalmology, Chhindwara Institute of Medical Sciences, Chhindwara, Madhya Pradesh, India; 3 Department of Physiology, Chhindwara Institute of Medical Sciences, Chhindwara, Madhya Pradesh, India; 4 Department of ENT, Chhindwara Institute of Medical Sciences, Chhindwara, Madhya Pradesh, India

**Keywords:** Rectal prolapse, modified Delorme's procedure, perineal repair, anal continence, elderly, surgical outcomes

## Abstract

Modified Delorme's Procedure is a safe and effective perineal approach to treat complete rectal prolapse, especially in the elderly and high-risk patients unfit for trans-abdominal surgery. Hence, a total of 65 patients (mean age 62.3 years; female: male ratio 6:1) underwent Modified Delorme's Procedure. The present prospective study of 65 patients with a mean age of 62.3 years, with a female to male ratio of 6:1, demonstrated a 92.3% success rate, with marked improvement in obstructed defecation and fecal continence on validated scales. Minimal postoperative complications included a recurrence rate of 7.7% and transient anal stenosis in 4.6%. The combination of mucosal stripping, muscular plication and adjunct postanal repair offers durable relief of symptoms with less morbidity than abdominal procedures.

## Background:

Rectal prolapse is an uncomfortable condition, primarily seen in the elderly, where a full-thickness protrusion of the rectum occurs through the anal canal. The incidence is notably higher in females, with a male-to-female ratio of approximately 1:6 and peaks between 50 and 60 years of age [1]. Patients with rectal prolapse typically present with symptoms of obstructed defecation, fecal incontinence and discomfort, severely impacting quality of life [2]. Although the etiology of rectal prolapse is multifactorial, ranging from pelvic floor dysfunction to chronic straining, management is a much-debated topic [3]. Traditionally, many authors have preferred transabdominal procedures, especially in healthy patients, due to lower recurrence rates and the potential for rectopexy [4]. The abdominal route may not be ideal in older adults or those with significant comorbidities due to the increased risk of perioperative morbidity [5]. On the other hand, perineal approaches, such as the Modified Delorme's Procedure, can be applied with advantages using local anesthesia or regional techniques and with less overall operative stress [6]. This modification not only addresses mucosal resection but also includes muscular plication and an adjunct postanal repair, both in correcting the anatomical defect and functional issues, particularly with anal sphincter weakness, going beyond mere mucosectomy [7]. The aim of the current study was to assess the safety and efficacy of the Modified Delorme's Procedure in patients who have complete rectal prolapse. This study seeks to provide information on the application of this surgical method in a clinical setting by documenting intraoperative parameters, postoperative complications and follow-up results systematically [8]. Further, the results are compared with historical data for traditional transabdominal approaches so that an integrated view of the best management plan for rectal prolapse is provided, especially for elderly patients. The study states that even though both techniques are viable options, it is the selection of the patient and the surgeon's expertise that determines the long-term success and improvement in quality of life [1, 3, 5 and 6]. In summary, this study investigates the Modified Delorme's Procedure as a safer alternative for patients suffering complete rectal prolapse through clearly defined procedural descriptions and postoperative outcomes that may significantly influence surgical practice in high-risk patient cohorts. Therefore, it is of interest to report the clinical outcomes and procedural details of the Modified Delorme's Procedure in the management of complete rectal prolapse in elderly and high-risk patients.

## Materials and Methods:

This retrospective study was conducted over five years (2018-2023) in the Department of Surgery at CIMS and District Hospital, Chhindwara, MP, after obtaining approval from the institutional ethical committee (Ref. No. CIMS/EC/2025/250). Informed consent was obtained from all patients following detailed counselling about the disease and potential complications. Adults and elderly patients were taken in this study and conditions such as pregnancy, prior anal surgery, neuropathy of pudendal nerve, fistula of the anus, sepsis, benign, or malignant anorectal lesions were excluded from the patients. Patients aged 80 years or more or suffering from vascular diseases, scleroderma, malnutrition, or coagulopathy were also excluded. All the patients had undergone a detailed history and clinical examination with laboratory investigations that included complete blood counts, coagulation profiles and biochemical tests. The preoperative workup included a proctoscopic examination and radiological evaluations such as defecography where indicated. Preoperatively, rectal enemas were used for bowel clearance and a third-generation cephalosporin was given prophylactically two hours before the procedure. The Modified Delorme's Procedure was done under general or spinal anesthesia with the patient in the lithotomy position. The prolapsed rectum was grasped externally and adrenaline saline was injected in the submucosal plane above the dentate line to allow dissection. A circumferential mucosal incision was done 1 cm proximal to the dentate line and mucosal dissection was extended up to the apex of the prolapse using electrocautery. The muscular layers were reapproximated with vicryl 2/0 sutures. Internally, mucosal stripping was done to mimic the external incision. A postanal repair was performed through a 7 cm incision behind the anal canal. Dissection of the intersphincteric plane was done and the internal sphincter was plicated with 3/0 vicryl sutures. The levator ani and external sphincter were sutured with prolene 2/0 and then skin closure was done. One week, three months, six months and one year postoperatively were followed and there was monitoring of the patient for signs of recurrence, anal stenosis, or impaired continence and also follow-up and follow-ups, as necessary.

## Results:

The study evaluated 65 consecutive patients (mean age: 62.3 years; range: 48-79 years) who underwent the Modified Delorme's Procedure for complete rectal prolapse. The female-to-male ratio was 6:1, with the majority of patients presenting with symptoms of obstructed defecation (76.9%) and fecal incontinence (53.8%). The median duration of symptoms was 3.2 years. Table 1 (see PDF) below provides a detailed breakdown of the demographic and clinical characteristics of the patients included in the study. It highlights the predominance of female patients (6:1 ratio) and the significant prevalence of symptoms like obstructed defecation (76.9%) and fecal incontinence (53.8%), with mean symptom duration of 3.2 years. Table 2 (see PDF) below provides an overview of intraoperative and early postoperative outcomes. The procedure demonstrated minimal operative challenges. The mean operative time was 85 ± 20 minutes, with an average blood loss of 75 mL. Intraoperative complications occurred in only 2 cases (3.1%). Postoperatively, patients required a mean hospital stay of 3.4 ± 1.2 days. Early complications were minor, including wound infections in 3 patients (4.6%). Table 3 (see PDF) below illustrates the functional and long-term outcomes of the procedure. At one year postoperatively, the recurrence rate was 7.7% (5 patients), primarily among those with severe baseline sphincter dysfunction. Functional outcomes demonstrated significant improvements, with the mean Wexner score improving from 14.2 preoperatively to 5.3 postoperatively (p < 0.01). Radiological evaluations showed enhanced anorectal angles and reduced residual intussusception in patients who underwent defecography. The study results, presented in Tables highlight the efficacy and safety of the Modified Delorme's Procedure. Table 1 (see PDF) shows a predominance of female patients (6:1 ratio) with a mean age of 62.3 years and symptoms such as obstructed defecation (76.9%) and fecal incontinence (53.8%). Table 2 (see PDF) demonstrates minimal complications, with a mean operative time of 85 minutes, average blood loss of 75 mL and a short hospital stay of 3.4 days. Table 3 (see PDF) reveals significant functional improvements, including a reduction in the mean Wexner score from 14.2 to 5.3 (p < 0.01), an 85% improvement in defecatory function and a low recurrence rate of 7.7%, emphasizing the procedure's success and durability.

## Discussion:

This study demonstrates that the Modified Delorme's Procedure is a safe and effective surgical option for complete rectal prolapse, especially in elderly and comorbid patients unsuitable for abdominal surgery [9]. With a high success rate of 92.3% and a low recurrence rate of 7.7%, our findings are in line with the existing literature supporting the perineal approach [10]. The procedure's minimally invasive nature, with a mean operative time of 85 minutes and an average hospital stay of less than four days, highlights its advantages in reducing physiological stress and facilitating faster recovery, making it ideal for frail patients [11]. Compared to the transabdominal approach, which is traditionally favored for its lower recurrence rates, the Modified Delorme's Procedure offers comparable efficacy with significantly lower morbidity [12]. The adjunct postanal repair in our technique further enhances anal sphincter function, improving continence and quality of life [13]. Recurrence in 5 patients was linked to severe preoperative sphincter weakness, underscoring the importance of thorough preoperative assessment and individualized patient selection [14]. A greater degree of constipation was observed in patients who had a later recurrence of the rectal prolapse [15]. This study highlights the need for a multicomponent approach using clinical, radiological and functional assessments, such as the Wexner score, to compare outcomes in surgical intervention. While both routes have their merits and drawbacks, Modified Delorme's Procedure leads to excellent outcomes in high-risk cohorts; however, randomized studies comparing these modalities might further define the treatment modalities for rectal prolapse in the future.

## Conclusion:

Modified Delorme's procedure is a safe, effective and minimally invasive option for complete rectal prolapse management in elderly patients or those with contraindications to abdominal surgery. This study shows significant functional improvements, low recurrence rates and minimal perioperative morbidity. These results support its position as a first-line surgical option for appropriately selected patients, thus providing a basis for wider clinical practice and further comparative studies.
